# The Automatic Methods Group *Newsletter*

**DOI:** 10.1155/S1463924696000211

**Published:** 1996


Journal of Automatic Chemistry, Vol. 18, No. 5, (September–October 1996) pp. 187–190

**AUTOMATIC METHODS GROUP**

Analytical Division
ROYAL SOCIETY OF CHEMISTRY

### The Automatic Methods Group *Newsletter*

The AMG Newsletter is published as part of and in collaboration with the *Journal of Automatic Chemistry*. Further information on the *Journal of Automatic Chemistry* can be obtained from Taylor & Francis Ltd, Rankine Road, Basingstoke, Hampshire RG24 8PR or e-mail:info@tandf.co.uk; worldwide web home page: http:||tandf.co.uk.

### Meeting Report

### *BP IV Automation and industrial process analysis*

*The following abstracts are from papers presented at the 'BP IV Automated process monitoring' meeting held at BP Hull on 22 May 1996. Whilst this meeting had a small attendance, the delegates had a very valuable tour of the BP facilities as well as listening to a series of well presented and helpful papers. This meeting was the fourth in a series of meetings organized in association with BP; the fifth is already being planned and will be held at BP Sunbury in May 1997.*

### Abstracts of papers presented

### At-plant gas chromatographic analysis

**Malcolm Cooper and John Green**
*BP Chemicals, Hull*

Of the several approaches to process analysis, at-plant analysis is proving to offer a number of significant advantages. Such systems involve placing analytical equipment adjacent to plant operations such that it can be used by operations staff as and when needed.

These developments have proved possible as a result of the advent of more reliable and flexibly programmable gas chromatographic systems. Consequently a gas chromatograph with autosampler can be programmed using a PC based control system to specify choice of GC method and calibration protocol. Operation of the analyser involves placing vials in the autosampler tray followed by pressing a single button. Different methods are accessed by selecting the position for the sample vial in the autosampler tray. Results are output to a printer or to a LIMS.

Operator intervention is reduced to a minimum whilst incorporating procedures to satisfy the varied requirements. Automatic system flushing allows analyses of crude and pure streams to be carried out on a single system, and check-samples ensure the integrity of the analyses.

Examples of the use of laboratory gas chromatographic equipment were described in at-plant situations together with a discussion of the advantages realized by such an approach.

### Some elements of on-line analysis

**J. R. P. Clarke**
*Department of Instrumentation & Analytical Science, UMIST, Manchester*

The need to survive leads to our requirement for clean air and water, good agriculture and food, health, energy and raw materials, adequate shelter, tools, security and transport. So a recent estimate that Western Europe spends 3% of its Gross Domestic Product on chemical measurements may not come as too great a surprise.

Analytical measurements are required in order to provide quantitative and qualitative information about chemicals. This information is needed in order that decisions can be made, for example, to control or optimize industrial products or plant, for peoples' health or safety and for the environment.

Analytical measurements are based upon an interacting three part system comprising:

(1) Sampling.
(2) Transduction of the energy used for the measurement to a (normally) electrical signal.
(3) Signal processing, to convert data into presentable information.

Before designing an on-line analysis system it is necessary to agree the end-user's aims. On discussion these may

0142-0453/96 $12·00 © 1996 Taylor & Francis Ltd change radically. The analytical check list is the foundation to the collection of pertinent information needed to plan what is to be done.

In this paper some trends in the interlinked elements of sampling, transduction and signal processing were discussed.

### Automated on-line process analysis

**Malcolm Crook (presented by Ron Belchamber)**
*Process Analysis and Automation, Farnborough*

The near-infrared (NIR) region of the spectrum has many properties that make it an ideal probe for on-line process analysis. The particular advantages that NIR confers over other spectral regions are:

(1) All organic compounds have characteristic NIR absorptions.
(2) Absorption bands are weak, which means that bulk constituents can be analysed over conveniently long optical pathlengths.
(3) Scattering from particulates has a smaller effect than in the UV and visible regions.
(4) Fibre optics transmit well in the near-infrared allowing measurements to be conveniently made.

The disadvantage of NIR is that the spectra are often poorly resolved with broad and difficult to assign bands. This requires multivariate calibration techniques to be used in most cases. The driving force behind the evolution of NIR has been the parallel development in chemometrics. These are statistical techniques that rely on developing calibration models based on training sets of data. The models predict constituent concentrations or other material properties directly from the spectra. Often you can completely avoid the intermediate step of determining chemical composition. For instance, a major application in the petroleum industry is the prediction of gasoline octane number directly from the NIR spectrum. The composition of the gasoline controls both the NIR spectrum and the octane number.

Where it is possible to make on-line measurements, there are many important benefits to be had over conventional sampling and laboratory analysis. Most notably, on-line analysis is much faster than any off-line procedure. To control a process, information must be obtained more rapidly than the process conditions change. To control a process, information must be obtained as rapidly as the process conditions change.

On-line NIR analysis is equally applicable to batch processes as it is to continuous processes. Monitoring of batch processes brings about the following advantages:

(1) Consistent product quality.
(2) Reduced wastage.
(3) Reduced batch times.
(4) Reduced exposure of personnel to harmful substances.

This presentation covered the principles of on-line NIR and the use of advanced mathematical techniques (CharmPLS) to resolve the answers from the complex data.

### Flow injection analysis in process analysis

**Peter R. Fielden**
*Department of Instrumentation and Analytical Science, UMIST, Manchester*

Viewed simply, flow injection analysis (FIA) is a means of handling and processing liquid samples for chemical analysis. Liquids are transported along small-bore (typically 0·5–1 mm i.d.) tubes and merged with reagents, diluents and standards in a controlled and reproducible way. The application of FIA to process analysis offers a number of advantages over alternative means of sample processing. At best it can be a simple, reliable, rapid and low-cost technique, conveniently configured for detection by optical, electrochemical or other instrumental measurement devices. Any wet chemical analytical procedure must offer low maintenance and low reagent consumption it it is to be viable for process analytical applications. The features of FIA as a process analysis interface were explored and illustrated:

(1) On-line stripping voltammetry for trace metal determination illustrated the versatility of FIA applied to the automation of a multi-stage analysis procedure.
(2) On-line toxicity measurement illustrated how a manually-intensive laboratory procedure may be realized in a FIA system for rapid toxicity assay, using luminescent bacteria.
(3) Hydrodynamic injection, within a FIA manifold demonstrated the possibility of chemical analysis with sample passage through components that have no moving parts. The possibility of further ruggedizing FIA for process analysis was discussed.

### Forthcoming Meeting

### Thermal Desorption—15 Years On

**A joint meeting of the Automatic Methods Group and the South East Region of the Analytical Division of the Royal Society of Chemistry, in cooperation with the Health & Safety Executive and sponsored by Perkin-Elmer. To be held at the Scientific Societies Lecture Theatre, London on Tuesday 17th December 1996.**

Sorption–desorption properties of a wide range of adsorbents have been studied for many years particularly by chromatographers. However, it was the need to develop a pre-concentration technique to measure the low levels of solvent vapour in ambient air, and proposed legislation controlling personal exposure, that led to the development of thermal desorption. The first commercial thermal desorption system was available in the early 80s and quickly became established as the preferred technique for monitoring VOCs in air. The aim of this meeting is to review the developments in thermal desorption techniques over the years and to examine the underlying theory. Forthcoming legislation will be examined and papers on a wide range of applications are to be presented illustrating the scope of the technique.

The meeting will be of interest to all users of thermal desorption systems and analysts who wish to monitor trace levels of VOCs in ambient air whether of consumer interest or for legislative purposes.

**Programme**

09.30 Thermal desorption, the real story
Dr Richard Brown, Health & Safety Executive, Sheffield

10.00 Theory of sorption, desorption
Dr Alan Braithwaite, Nottingham Trent University
Dr Theo Haftenscheid, TNO, Netherlands

11.00 Coffee

11.30 EU Ambient Air Directive, impact and implementation
Dr Emile De Sager, ISPRA, Italy

11.50 Calibration and standards, QA/QC, how not to do it

Bill Boyle, BP International, Sunbury

12.10 Discussion

12.20 Lunch

13.30 ATD as an autosampler
Liz Wolfenden, Perkin-Elmer, USA

14.00 Purge and trap techniques, and applications using the ATD
Dr Alex Bianci, Esso, Fawley

14.20 Terpenes in ambient air, chemotaxonomy of plants using headspace and ATD
Dr John Twibell, Elsworth Herbs, Cambridge

14.40 On-line ATD to monitor ozone precursors
Greg Johnson, Perkin-Elmer, Beaconsfield

15.10 Monitoring PAHs from road traffic
Ian Rillie, Road Transport Research Laboratory, Bracknell

15.30 VOCs in alveolar air, bacterial signatures and occupational exposures
John Cocker, Health and Safety Executive, Bootle

15.50 Tea

16.15 Close of conference

The registration fee, which includes lunch and all refreshments, is £100 for members of the Royal Society of Chemistry and £140 for non-members of the Society. Students and retired members may register for a fee of £60.

Further information, and a registration form, may be obtained from Dr R. Narayanaswamy at DIAS, UMIST, PO Box 88, Manchester M60 1QD; Tel: 0161 200 4891/4885; fax: 0161 200 4911; e-mail: ramaier.narayanaswamy@umist.ac.uk.

A limited number of student bursaries are available. Please contact Dr Narayanaswamy for details.

**(Please turn over for Automatic Methods Group Questionnaire.)**

